# Antioxidant and anti-inflammatory activities of selected medicinal plants and fungi containing phenolic and flavonoid compounds

**DOI:** 10.1186/1749-8546-7-26

**Published:** 2012-11-24

**Authors:** Patricia Diaz, Sang Chul Jeong, Samiuela Lee, Cheang Khoo, Sundar Rao Koyyalamudi

**Affiliations:** 1 Centre for Complementary Medicine Research, University of Western Sydney, Locked Bag 1797, Penrith South DC, NSW 1797, Australia; 2 School of Science and Health, University of Western Sydney, Locked Bag 1797, Penrith South DC, NSW 1797, Australia

## Abstract

**Background:**

This study aims to determine the relationship between the antioxidant and anti-inflammatory activities of the thirteen herbs and two fungi extracts, and their total phenolic and flavonoid contents.

**Methods:**

Antioxidant activities were evaluated by four assays: an antioxidant activity assay using *Saccharomyces cerevisiae*, a DPPH ((2, 2-diphenyl-1-picrylhydrazyl) assay to assess free radical scavenging, an assay assessing ferrous ions or iron (II) chelating ability, and a ferric reducing antioxidant power (FRAP) assay. Total phenolic and flavonoid contents were determined using the Folin-Ciocalteu and aluminium chloride methods, respectively. Anti-inflammatory activities were determined by measuring the inhibition of nitric oxide and TNF-α production in lipopolysaccharide- and interferon-γ-activated J774A.1 macrophages. Their cytotoxicities against macrophages were determined by MTT assay.

**Results:**

A positive linear correlation between antioxidant activities and the total phenolic and flavonoid content of the plant extracts was found. The plant extracts with high phenolic and flavonoid content also exhibited significant anti-inflammatory activity with good cell viability.

**Conclusion:**

The selected herbs could be a rich source of antioxidants and free radical scavenging compounds. The levels of phenolic and flavonoid compounds were correlated with the antioxidant and anti-inflammatory activities of the extracts from the herbs.

## Background

Reactive oxygen species (ROS) include free radicals, *e.g.*, superoxide (O_2_^●**-**^) and the hydroxyl (OH^●^), hydroperoxyl (OOH^●^), peroxy (ROO^●^) and alkoxy (RO^●^) radicals, and non-free radicals, *e.g.*, hydrogen peroxide (H_2_O_2_) and hypochlorous acid (HOCl), which are constantly produced in the human body during cell metabolism [1]. Others are reactive nitrogen species (RNS) consisting of nitric oxide (NO^●^), peroxynitrite (ONOO^●^) and nitrogen dioxide (NO_2_). Free radicals are important in the regulation of signal transduction, gene expression and activation of receptors [2]. However, an excess of free radicals is toxic to almost every biological molecule in living cells [3], and can cause oxidative damage to functional macromolecules such as DNA, proteins, and lipids if not eliminated quickly [4]. Excess generation of free radicals can lead to many diseases such as age-related disorders, cancer, atherosclerosis, neurodegenerative diseases and inflammation [5,6]. Antioxidant compounds from plants can minimize the generation of free radicals [6,7] and alleviate diseases caused by oxidative stress [8,9]. The phenolics and flavonoids of medicinal herbs contribute to the antioxidant activities of plants [4,6,10], and act as anti-inflammatory agents [11]. Production of proinflammatory molecules such as TNF-α and nitric oxide (NO) can modulate inflammation. These inflammatory molecules react with free radicals; for example, NO reacts with O_2_^●**-**^ to produce ONOO^●^, which can cause irreversible damage to cell membranes, leading to cell death and tissue damage [12].

Thirteen medicinal plants and two fungi (Table 1) were chosen in this study for the measurement of antioxidant and anti-inflammatory activities. These plants and fungi are currently used by Chinese medical practitioners, in clinical studies, or both (Table 1). The antioxidant and anti-inflammatory activities of selected thirteen TCM plants and two fungi were measured systematically with a series of assays, as well as their relationship with the total phenolic and flavonoid contents.

**Table 1:** List of medicinal herbs

**Herbs No.**	**Plant name**	**Family name**	**Chinese name**	**Herb part used**	**Medicinal use**
H1	*Baccharis genistelloides *(Lam.) Pers.	*Asteraceae*	*Zhong jie feng*	leaf	Gastritis, indigestion, heartburn, gallbladder stones, constipation, jaundice, diabetes, urinary tract infections (UTI), spleen dysfunction, liver disorders, fever and worms.^1^
H2	*Physalis alkekengi *L.	*Solanaceae*	*Jin Deng Long*	fruit	Sore throat and hoarseness of voice; cough with yellow sticky sputum; dysuria; external use for pemphigus and eczema.^2^
H3	*Taraxacum mongolicum *Hand-Mazz.	*Compositae*	*Pu Gong Ying*	leaf, flower and stems	Boils and sores, mastitis lymphadenitis, inflammation of eyes, sore throat, lung abscess, appendicitis, jaundice caused by damp-heat, urinary infection with difficult painful urination.^2^
H4	*Lasiosphaera fenzlii *Reich	*Lycoperdaceae*	*Ma Bo*	fungus	Sore throat, cough and hoarseness caused by wind-heat in the lung; epistaxis and traumatic bleeding in external application.^2^
H5	*Belamcanda chinensis *L.	*Iridaceae*	*She Gan*	root	Sore throat, cough and dyspnoea with expectoration of copious phlegm due to accumulation of toxic heat, phlegm and fire.^2^
H6	*Tinospora capilipes *Gagnep.	*Menispermaceae*	*Jin Guo Lan*	root	Swelling and pain of the throat, carbuncles and boils; diarrhoea, dysentery; epigastric hot pain.^2^
H7	*Artemisia argyi *Levl. & Vant.	*Compositae*	*Ai Ye*	leaf	Cold pain in the lower abdomen; menstrual disorders caused by cold; infertility; spitting of blood, epitasis, uterine bleeding in pregnancy, excessive menstrual flow or prolonged menstruation; external use for itching.^2^
H8	*Morus alba *L.	*Moraceae*	*Sang Ye*	leaf	Common cold due to -wind-heat, dry cough due to heat in the lung dizziness, headache, inflammation of the eye, blurred vision.^2^
H9	*Prunella vulgaris *L.	*Labiatae*	*Xia Ku Cao*	fruit-spike	Inflammation of the eyes, ophthalmalgia at night, headache and dizziness; scrofula, goitre, mastitis with swelling and pain, hyperplasia of breast; hypertension.^2^
H10	*Lophatherum gracile *Brongn	*Gramineae*	*Dan Zhu Ye*	leaf	Restlessness and thirst in febrile diseases, ulcers of the tongue and the mouth, swelling and pain of gingiva, pharyngolaryngitis, oliguria, phlegmonosis and odynuria, dysuria with dark urine, and painful urination.^2^
H11	*Cordyceps militaris *L.	*Clavicipitaceae*	*Dong Chong Xia Cao*	fungus	Chronic cough and asthma; haemoptysis in phthisis; impotence and seminal emission with aching of loins and knees.^2^
H12	*Scutellaria baicalensis *Georgi.	*Labiatae*	*Huang Qin*	root	Oppressed feeling in the chest, nausea and vomiting in epidemic febrile diseases caused by damp-heat or summer-heat; feeling of stuffiness in the abdomen, acute dysentery or jaundice caused by damp-heat; cough due to heat in the lung; high fever with dire thirst; spitting of blood and epitasis due to heat in blood; carbuncles and sores; threatened abortion.^2^
H13	*Platycodon grandiflora *(Jacq.) A.DC.	*Campanulaceae*	*Jie Geng*	root	Cough with much phlegm, sore throat, hoarseness; pulmonary abscesses with pus running abscess difficult to burst after suppuration.^2^
H14	*Epimedium brevicornum *Maxim	*Berberidaceae*	*Yin Yang Huo*	leaf	Impotence, seminal emission, weakness of the limbs; rheumatic or rheumatoid arthralgia with numbness and muscle contracture; climacteric hypertension.^2^
H15	*Conyza Bonariensis *(L.) Cronquist	*Asteraceae*		whole plant	Diuretic.^1^

^1^Campos, N (2006). Chapter: Carqueja. Aprendendo com a Mae Terra – Plantas Medicinais, Aromáticas e Condimentares. Sao Paulo, Brazil. Arte & Ciencia. p59.

Unless specified, information on medicinal function was obtained from China Pharmacopoeia Committee (2005).

^2^Pharmacopoeia of the People’s Republic of China (Vol. 1). Beijing: China Chemical Industry Press.

## Methods

### Plant materials

The dried plant materials were obtained from the Beijing Tong Ren Tang Chinese Herbal Medicine shop (Sydney, Australia). A voucher specimen of each sample has been deposited in the Herbal Analysis Laboratory, University of Western Sydney. The scientific names and family names of the herbs are shown in Table 1. The samples were ground to a fine powder in a grinder before extraction.

### Chemicals and reagents

Gallic acid, quercetin, 2, 2-diphenyl-1-picrylhydrazyl (DPPH), dimethyl sulfoxide (DMSO), sodium carbonate, aluminium chloride, sodium nitrate, sodium hydroxide, H_2_O_2_, Folin-Ciocalteu (F-C) reagent, ascorbic acid, 95% ethanol, bovine serum albumin (BSA), lipopolysaccharide (LPS: *E. coli* serotype 0127:B8), EDTA, N-(1-1-napthyl) ethylenediamine dihydrochloride, penicillin G sodium benzyl, resazurin sodium 10%, streptomycin, sulfanilamide, tetramethyl benzidine (TMB) and trypan blue were purchased from Sigma (Australia) and Lomb Scientific Pty Ltd. (Australia). Antibiotics, Dulbecco’s modified Eagle’s medium (DMEM), foetal bovine serum (FBS) and glutamine were purchased from GIBCO. Interferon-γ (murine) and tumour necrosis factor-α (TNF-α) enzyme-linked immunosorbent assay (ELISA) kits were purchased from BD Bioscience (San Jose, CA, USA).

### Preparation of water extracts

Sample powder (3 g) was autoclaved with 30 mL deionised water at 121°C for 1 h. The extracted samples were centrifuged at 10,447 × *g* (Sorvall Ultra pro 80, Kendro Instruments Australia Pty, Ltd) for 20 min and the supernatants were transferred to a 50-mL volumetric flask. The residues were rinsed two more times, and the pooled extract was adjusted to 50 mL with deionised water. The samples were stored at −20°C until analysis of phenolic and flavonoid contents.

### Preparation of ethanol extracts

Sample powder (3 g) was extracted with 30 mL of 95% ethanol in a water bath at 70°C for 6 h. The extracted samples were centrifuged and the supernatants were transferred to a 50-mL volumetric flask. The residues were rinsed two more times and the pooled extracts were adjusted to 50 mL with 95% ethanol. The samples were stored at −4°C until analysis of phenolic and flavonoid content. All water and ethanol extracts were filtered through a 0.45-μm nylon filter before analysis.

### Determination of total phenolic and flavonoid content

The total phenolic content was determined according to the Folin-Ciocalteu (F-C) colorimetric method [13]. Briefly, 50 μL of sample and 50 μL of F-C reagent were pipetted into an Eppendorf tube. The contents were vortexed for 10 s and then left to stand at room temperature for 2 min before the reaction was stopped by adding 500 μL of 5% (w/v) sodium carbonate solution and 400 μL of distilled water, and the volume was adjusted to 1 mL. The mixture was then vortexed and incubated at 45°C for 30 min before cooling rapidly with ice. The absorbance of the solution was measured at 760 nm. Gallic acid concentrations ranging from 25 to 300 μg/mL were prepared and a calibration curve was obtained using a linear fit [14]. The samples were analysed in duplicate.

The total flavonoid content was determined according to the aluminium chloride method [15]. Briefly, 0.5 mL of sample and 300 μL of NaNO_2_ (1:20 w/v) were pipetted into a test tube and the contents were vortexed for 10 s and left to stand at room temperature for 5 min. After standing, 300 μL of AlCl_3_ (1:10 w/v), 2 mL of NaOH (1 M) and 1.9 mL of distilled water were added to the reaction mixture, which was then vortexed for 10 s, and the absorbance was measured at 510 nm. Quercetin concentrations ranging from 0 to 1200 μg/mL were prepared and a standard calibration curve was obtained using a linear fit. The samples were analysed in duplicate.

### Free radical DPPH scavenging assay

The DPPH assay was carried out according to procedures of Brand-William *et al.*[16], with minor modifications. Different volumes (10, 20, 30, 40, 50, 60, 70, 80, 90, and 100 μL) of ethanol and water extracts were mixed with DPPH radical in methanol (2.2 mg/L, 200 μL) in a 96-well microplate. The final volume of each well was made up to 300 μL by adding the appropriate amount of methanol. The mixture was shaken gently on a microplate reader (Bio-Rad, Hercules, CA ,USA) and the absorbance at 515 nm was measured every 2 min for 30 min or until the absorbance reached its maximum value. The DPPH concentration in the reaction medium was calculated from a calibration curve derived from serial dilution of the DPPH standard. The control (containing all reagents except the test compound) and standards were subject to the same procedure. The free radical scavenging activity was expressed as the percentage inhibition of free radical generation by the sample, and calculated using the following formula:

(1) $$I\left(\%\right)\;\text{of DPPH radical scavenging effect}=\left[\left({\text{A}}_{\text{control}}–{\text{A}}_{\text{sample}}\right)/{\text{A}}_{\text{control}}\right]\times 100$$

where A_control_ is the absorbance of the control, and A_sample_ is the absorbance of the sample at 515 nm. The samples were analysed in triplicate.

### Assay for scavenging activity using *Saccharomyces cerevisiae*

The antioxidant activities of the ethanol and water extracts were also measured according to a *S. cerevisiae*-based high throughput assay [17]. *S. cerevisiae* BY4743 was cultured overnight in a 50-mL volume by inoculation of a single colony. The culture was then diluted to an optical density at 600 nm (OD_600_) of 0.2 in media, and 180 μL of the culture broth was added to each well in a 96-well microtitre plate to which 10 μL per well of each herbal extract had been added in duplicate. H_2_O_2_ (10 μL, 32% v/v) was added to give a final concentration of 4 mM. The initial OD_600_ reading was taken using a microplate reader (Multiskan EX, Thermo Electron, USA), and the plates were then incubated in an incubator at 30°C with shaking at 750 rpm. Yeast growth was monitored by reading the OD_600_ until 20 h. Ascorbate was used as the positive control. The samples were analysed in duplicate.

### Ferrous ion-chelating effect

The ferrous ion-chelating effects of the extracts were estimated according to the method of Chua *et al.*[18]. Briefly, 740 μL of methanol and 200 μL of sample were incubated with 20 μL of FeCl_2_ solution (2 mM). The reaction was initiated by adding 40 μL of ferrozine (5 mM) into the mixture, which was then allowed to stand at ambient temperature for 10 min. The absorbance of the reaction mixture was measured at 562 nm. Distilled water instead of ferrozine solution was used as a blank, to correct for any unequal colour of the sample solution. EDTA-Na_2_ was used as a reference standard. The ferrous ion-chelating ability was calculated as follows:

(2) $$\text{Ferrous ion}\text{chelating ability}\left(\%\right)=\left[{\text{A}}_{\text{control}}-\left({\text{A}}_{\text{sample}}-{\text{A}}_{\text{blank}}\right)\right]/{\text{A}}_{\text{control}}\times 100$$

where A_control_ is the absorbance of the control, A_sample_ is the absorbance of the sample or standard, and A_blank_ is the absorbance of the blank.

### Ferric-reducing antioxidant power assay

The FRAP of the extracts was tested according to Oyaizu [19]. Different concentrations of the samples (1 mL) and the reference chlorogenic acid were added to 2.5 mL of phosphate buffer (0.1 M, pH 6.6) and 2.5 mL of potassium ferricyanide (1.0%, w/v). Each mixture was incubated at 50°C for 20 min and 2.5 mL of trichloroacetic acid (10%) was added. The mixture was shaken vigorously, and the solution (2.5 mL) was mixed with 2.5 mL of distilled water and 0.5 mL of FeCl_3_ (0.1%, w/v). After a 30 min incubation, absorbances were read at 700 nm. Each analysis was carried out in duplicate. Increased absorbance of the reaction mixture indicates stronger reducing power.

### Maintenance, preparation and activation of J774A.1 macrophages

J774A.1 macrophages were incubated in 50-mL flasks in DMEM containing 10% FBS supplemented with antibiotics (1%) and glutamine (1%). The cells were maintained in 5% CO_2_ at 37°C, with the media being replaced every 3 days. Once the cells had grown to confluence in the culture flask, they were removed using a rubber policeman. The cell suspension was concentrated by centrifugation for 5 min at 180 × *g*, the supernatant was removed and the pellet was resuspended in a small volume of fresh DMEM (with 1% antibiotics and 10% FBS). The cell density was estimated using a Neubauer counting chamber (Neubauer Precicdor HBG, Germany). Cell concentration was adjusted using DMEM (with 1% antibiotics and 10% FBS) to obtain 75,000 cells/well, after which 180 μL of the cell suspension was dispensed into the 60 inner wells of a 96-well plate. Sterile distilled water was added to the outer row of wells. For assays of the extracts, 20 μL of the extract solution was added with or without activator (1 μg/mL LPS and 10 U/mL IFN-γ). In most cases, the maximum dose of extract used was 1 mg/mL with a minimum of five doses made by serial dilution. The cells were incubated for 24 h at 37°C and 5% CO_2_. Cells with media alone were used as negative controls and activated cells were used as positive controls.

### Griess assay

NO level was determined with the Griess reagent [20], which quantifies nitrite, one of the stable reaction products. Briefly, the supernatant was mixed with an equal volume of Griess reagent (0.1% *w/v* naphthyl ethylenediamine and 1% *w/v* sulfanilamide in 5% *v/v* phosphoric acid) in the wells of a 96-well flat-bottomed microtitre plate. After standing for 5 min at room temperature, the absorbance was measured at 550 nm using a Bio-Rad microplate reader. The remaining supernatant was removed from each well for the TNF-α assay by ELISA.

### MTT assay

Cell viability was assessed using a modified MTT assay [21]. Briefly, cells (7×10^5^ cells/well) were seeded in a 96-well plate and treated with 20 μl of sample extract. After incubation for 24 h, the supernatant was replaced with 200 μL of media with MTT solution (500 μg/mL). After further 4 h incubation at 37°C, 200 μL of DMSO was added to each well to solubilise the deposited formazan. The optical density of each well was measured at 490 nm by a microplate reader.

### TNF-α determination by ELISA

A sandwich ELISA method was used as described previously [22] to determine the TNF-α concentration. The capture antibody was used with 0.1 M sodium carbonate buffer (pH 9.5). Serial dilutions of TNF-α standard from 0 to 1000 pg/mL in diluent (10% BSA in PBS) were used as the internal standard. TNF-α was detected using a biotinylated secondary antibody and an avidin peroxidase conjugate with TMB as the detection reagent. After 30 min, the reaction was stopped by 2 M sulfuric acid and the absorbance was measured at 450 nm.

### Data presentation and analysis

The results were expressed as mean ± standard deviation (SD), calculated from duplicate determinations and the linear relationship was visually determined. The significance of differences among groups of data were determined with one-way analysis of variance (ANOVA) by using statistical software SPSS 18.0 for Windows (IBM, USA) and Duncan’s multiple range test [23]. The threshold for statistical significance was *P* < 0.05.

## Results and discussion

### Total phenolic and flavonoid contents

The yields of the hot water and ethanol extracts varied widely (Table 2). The yield of the water extracts (13.9–328.9 mg/g) was higher than that of the ethanol extracts (3.3–22.3 mg/g). The total phenolic and flavonoid contents of the extracts was measured using the F-C reagent and aluminium chloride methods, respectively. The results obtained with the water and ethanol extracts are presented in Tables 3 and 4, respectively.

**Table 2:** Dry weights of hot water extracts and ethanol extracts of the herbs

**No**	**Herbs**	**Hot water extract (mg/g)**	**Ethanol extract (mg/g)**
H1	*Baccharis genistelloides*	92.8	12.9
H2	*Physalis alkekengi*	134.7	10.0
H3	*Taraxacum mongolicun*	328.9	10.5
H4	*Lasiosphaerae seu calvatia*	13.9	4.1
H5	*Belamcandae chinensis*	156.9	15.1
H6	*Tinospora capilipes*	123.6	5.1
H7	*Artemisiae argyi*	24.9	3.3
H8	*Morus alba*	246.2	14.4
H9	*Prunella vulgaris*	60.8	4.9
H10	*Lophatherum gracile*	35.1	22.3
H11	*Cordyceps militaris*	306.1	11.2
H12	*Scutellaria baicalensis*	210.0	10.6
H13	*Platycodon grandiflora*	236.4	11.2
H14	*Epimedium brevicornu*m	18.3	13.1
H15	*Conyza bonariensis*	224.8	10.2

**Table 3:** Antioxidant activities of hot water extracts of the herbs

**Herbs (Hot water extract)**	**Scavenging activity on yeast**^**d**^	**Phenol content (GAE μg/mg)**	**Flavonoid content (QE μg/mg)**	**DPPH *I*(%)**^**a**^	**Ferrous ion-chelating ability**		**Ferric-reducing antioxidant power (CPE**^**c **^**mg/g)**
					**%**^**b**^	**EDTA equivalent (μg/mL)**	
H1	+	51.24 ± 0.13	200.08 ± 10.18	61.95 ± 1.25	70.85 ± 0.23	44.60 ± 0.16	53.31 ± 0.11
H2	+	51.80 ± 0.39	98.62 ± 2.83	72.57 ± 0.00	31.56 ± 0.35	18.85 ± 0.24	34.78 ± 0.14
H3	+	42.63 ± 0.00	146.88 ± 3.96	69.91 ± 0.00	20.21 ± 0.08	12.74 ± 0.05	50.91 ± 0.25
H4	+	57.26 ± 0.00	78.12 ± 1.27	63.27 ± 0.63	61.70 ± 1.27	33.15 ± 0.87	31.08 ± 0.14
H5	+	21.80 ± 0.71	32.02 ± 7.92	10.62 ± 1.25	48.98 ± 0.17	31.25 ± 0.12	23.31 ± 0.18
H6	+	12.69 ± 0.08	14.52 ± 3.25	11.50 ± 0.00	21.79 ± 0.13	11.86 ± 0.09	12.46 ± 0.18
H7	+	137.35 ± 0.13	532.48 ± 8.49	65.04 ± 0.63	143.50 ± 2.11	83.23 ± 1.45	56.72 ± 0.57
H8	+	47.91 ± 0.39	180.88 ± 5.66	66.37 ± 6.26	18.77 ± 0.34	11.36 ± 0.23	49.23 ± 0.21
H9	+	100.04 ± 0.52	79.12 ± 2.69	52.65 ± 30.66	106.16 ± 1.37	65.87 ± 0.94	103.92 ± 1.13
H10	+	68.19 ± 0.52	170.88 ± 2.83	58.41 ± 0.00	138.03 ± 1.20	83.98 ± 0.83	42.71 ± 0.18
H11	+	23.07 ± 0.20	26.52 ± 11.46	50.88 ± 1.88	22.15 ± 0.10	14.01 ± 0.07	20.98 ± 0.28
H12	+	93.56 ± 0.26	33.62 ± 5.09	76.99 ± 0.00	26.05 ± 0.30	16.09 ± 0.21	74.43 ± 0.35
H13	+	5.21 ± 0.03	9.92 ± 4.95	7.08 ± 6.26	13.92 ± 0.29	7.94 ± 0.20	2.96 ± 0.18
H14	-	101.89 ± 0.52	265.28 ± 9.62	76.11 ± 2.50	241.06 ± 2.30	144.82 ± 1.58	84.02 ± 0.99
H15	-	200.04 ± 0.52	276.48 ± 6.22	69.91 ± 2.50	15.18 ± 0.11	9.46 ± 0.07	54.36 ± 0.04

Data are presented in mean ± SD.

^a^The ratio of inhibition of free radical by DPPH in percent (*I*(%))as follows; *I *(%) = [(A_control _- A_sample_)/A_control_] × 100.

^b^Ferrous ion-chelating ability as follows; % = [A_control_-(A_sample_-A_blank_)] /A_control _× 100 (Chelating effect of EDTA (50ug/mL) as positive control is 100%).

^c^Ferric reducing antioxidant power was expressed in milligram of chlorogenic acid power (CPE) per gram of dry weight.

^d^Yeast oxidative stress was measured on the basis of survival of yeast cells (yeast growth) after treatment of H_2_O_2_, +: active; -: not active.

**Table 4:** Antioxidant activities of ethanol extracts of the herbs

**Herbs (Ethanol extract)**	**Scavenging activity on yeast**^**d**^	**Phenol content (GAE μg/mg)**	**Flavonoid content (QE μg/mg)**	**DPPH *I*(%)**^**a**^	**Ferrous ion-chelating ability**		**Ferric-reducing antioxidant power (CPE**^**c **^**mg/g)**
					**%**^**b**^	**EDTA equivalent (μg/mL)**	
H1	+	95.78 ± 0.26	77.32 ± 5.80	81.86 ± 0.63	15.43 ± 0.24	7.67 ± 0.17	79.23 ± 0.21
H2	+	53.09 ± 0.13	120.02 ± 2.55	51.77 ± 5.63	-	-	36.58 ± 0.28
H3	+	36.98 ± 0.65	32.22 ± 1.41	57.96 ± 4.38	16.65 ± 0.23	8.08 ± 0.16	31.48 ± 0.28
H4	-	28.93 ± 1.57	62.32 ± 0.99	20.70 ± 8.00	99.84 ± 3.48	60.72 ± 2.40	7.41 ± 0.32
H5	+	56.80 ± 0.13	53.32 ± 1.56	30.58 ± 9.46	8.78 ± 0.10	4.76 ± 0.07	14.08 ± 0.49
H6	+	15.15 ± 0.05	46.52 ± 0.14	22.04 ± 7.40	73.19 ± 0.28	43.49 ± 0.19	10.43 ± 0.07
H7	+	78.56 ± 0.52	59.62 ± 1.41	72.71 ± 6.46	28.81 ± 1.21	10.92 ± 0.83	58.18 ± 0.14
H8	-	21.43 ± 0.39	29.02 ± 3.11	36.85 ± 8.31	3.44 ± 0.06	0.92 ± 0.04	10.13 ± 0.07
H9	-	34.85 ± 0.26	194.02 ± 13.29	67.35 ± 8.89	-	-	43.26 ± 0.18
H10	+	161.15 ± 0.52	449.6 ± 14.14	238.94 ± 13.77	11.82 ± 0.86	-	39.61 ± 0.11
H11	+	21.70 ± 0.26	17.62 ± 1.41	4.42 ± 2.50	42.86 ± 0.58	26.23 ± 0.40	7.13 ± 0.14
H12	-	166.15 ± 0.26	117.82 ± 1.13	81.42 ± 0.00	38.28 ± 0.40	22.65 ± 0.28	87.52 ± 0.02
H13	-	8.60 ± 0.05	36.02 ± 0.57	2.65 ± 2.50	-	-	3.56 ± 0.11
H14	-	36.61 ± 0.39	35.12 ± 0.99	54.42 ± 3.13	38.66 ± 0.02	23.41 ± 0.01	14.16 ± 0.11
H15	+	56.61 ± 0.39	73.42 ± 3.96	75.66 ± 3.13	13.81 ± 0.24	7.71 ± 0.17	55.33 ± 0.21

Data are presented in mean ± SD.

^a^The ratio of inhibition of free radical by DPPH in percent (*I*(%))as follows; *I *(%) = [(A_control _- A_sample_)/A_control_] × 100.

^b^Ferrous ion-chelating ability as follows; % = [A_control_-(A_sample_-A_blank_)] /A_control _× 100.

^c^Ferric reducing antioxidant power was expressed in gram of chlorogenic acid power (CPE) per milligram of dry weight.

^d^Yeast oxidative stress was measured on the basis of survival of yeast cells (yeast growth) after treatment of H_2_O_2_, +: active; -: not active.

### Water extracts

**Figure 1:**
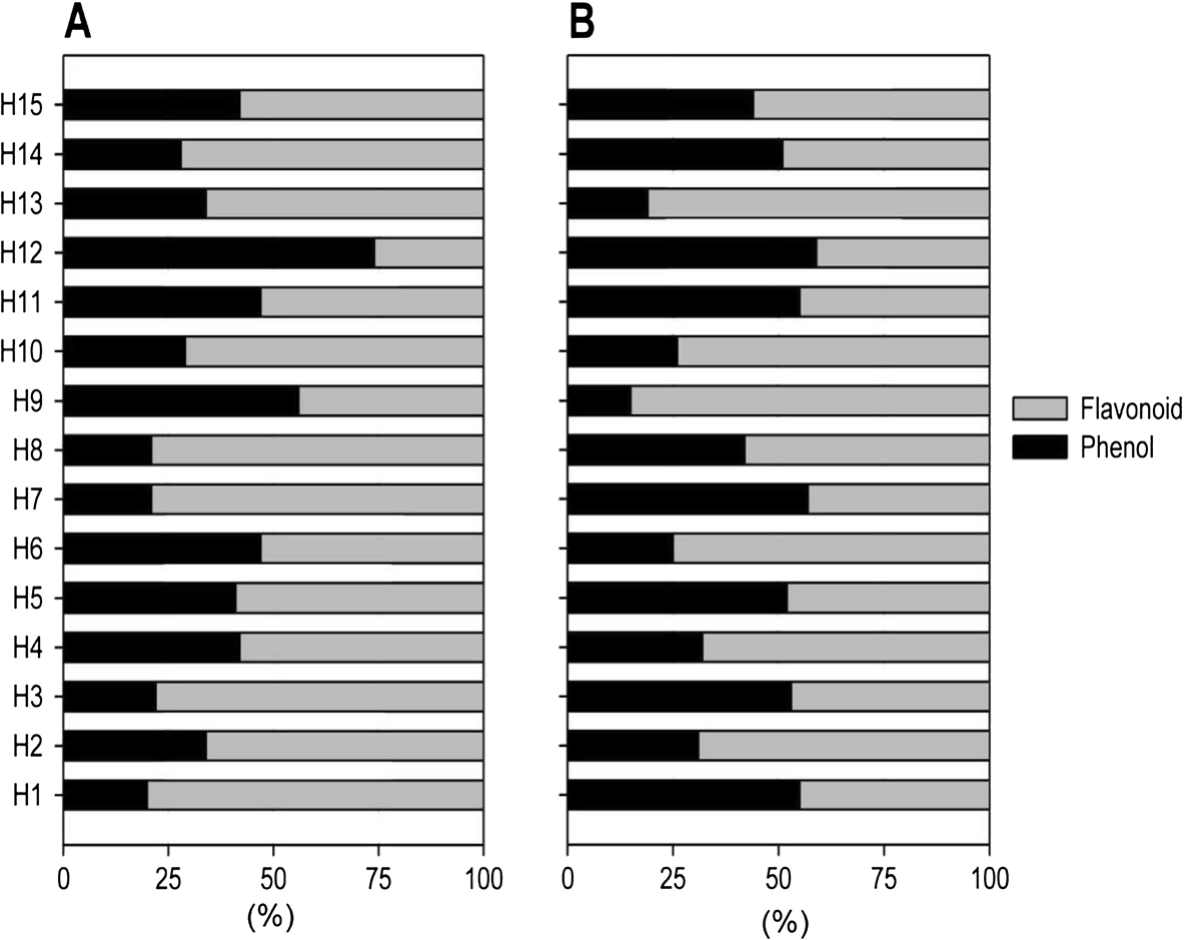
**Proportional relation (%) of flavonoid content to phenolic content in the herbal extracts. A**: Hot water extracts. **B**: Ethanol extracts.

The highest phenolic content was found in *C. bonariensis* (H15: 200.0 μg/mg), followed by *A. argyi* (H7: 137.3 μg/mg), *E. brevicornum* (H14: 101.9 μg/mg) and *P. vulgaris* (H9: 100.0 μg/mg), while the lowest content was found in *P. grandiflora* (H13: 5.21 μg/mg). In the other herbs the total phenolic content ranged 12.7 to 93.5 μg/mg. The flavonoid content varied greatly from 9.92 to 532.48 μg/mg (Table 3). The highest flavonoid content was found in *A. argyi* (H7: 532.48 μg/mg) followed by *C. bonariensis* (H15: 276.48 μg/mg), *E. brevicornum* (H14: 265.28 μg/mg), *B. genistelloides* (H1: 200.08 μg/mg) and *M. alba* (H8: 180.88 μg/mg). The water extracts contained a higher proportion (≥ 50%) of flavonoids than phenolics, except *S. baicalensis*, which contained 75% phenolic compounds (Figure 1A).

### Ethanol extracts

The flavonoid content of the herbs was in the range 17.6–449.6 μg/mg, with large variations between the plants (Table 4). The highest flavonoid content was found in *L. gracile* (H10: 449.6 μg/mg extract) followed by *P. vulgaris* (H9: 194.02 μg/mg extract), *P. alkekengi* (H2: 120.02 μg/mg) and *S. baicalensis* (H12: 117.82 μg/mg); while the lowest was in *C. militaris* (H11: 17.62 μg/mg). Phenolics and flavonoids neutralize free radicals by donating a hydrogen atom or an electron, and chelate metal ions [24]. The ethanol extracts contained a higher proportion of flavonoids than phenolics (Figure 1B).

### Antioxidant activities

The antioxidant activities of the water and ethanol extracts in this study were evaluated using an antioxidant assay for scavenging activity with *S. cerevisiae*, a DPPH free radical scavenging assay, an assay assessing the iron (II) chelating ability, and a FRAP assay. As presented in Table 3, thirteen samples showed antioxidant activity in the yeast-based assay that utilises the oxidative stress-mediated cell cycle arrest to reveal the oxidant scavenging and intracellular antioxidant activity. Most of the water and ethanol extracts (fourteen samples out of fifteen) displayed significant antioxidant activity as measured by the DPPH method. The results from the antioxidant assay suggested that the water extracts showed higher activity than the ethanol extracts in general. The percentage inhibitions of the free radical DPPH were in the range 7.08–69.91% for the water extracts and 2.65–238.94% for the ethanol extracts. Significant free radical scavenging activities (≥ 50%) were observed in all water extracts, except for those of *B. chinensis* (H5), *T. capilipes* (H6) and *P. grandiflora* (H13), in which the inhibition of DPPH was less than 10%. The highest inhibition of DPPH activity was found in the ethanol extract of *L. gracile* (H10: 238.94%). The free radical scavenging activities were very low in the ethanol extracts of *C. militaris* (H11: 4.4%) and *P. grandiflora* (H13: 2.6%). The herbs displayed antioxidant activities owing to a combination of their total phenolic and flavonoid content as reported in other studies [6,10].

The ferrous ion/iron (II) chelating abilities of the samples are summarised in Tables 3 and 4. Iron is the chief peroxidant and is able to generate lipid peroxidation through the Fenton reaction or by accelerating the dissociation of lipid hydroperoxides to their respective peroxy and alkoxy radicals [25]. The ability of the extracts to bind Fe^2+^ in the presence of ferrozine was compared with that of EDTA [26]. The water extracts had a higher Fe^2+^ chelating effect than the ethanol extracts. The water extracts of *A. argyi* (H7)*, P. vulgaris* (H9)*, L. gracile* (H10) and *E. brevicornum* (H14) exhibited 143.5, 106.2, 138.0 and 241.1% chelation of Fe^2+^ equivalent to 83.2, 65.9, 84.0 and 144.0 μg/mL EDTA, respectively. The highest percentage chelating capacity of the ethanol extracts was found in *L. Seu calvatia* (H4: 99.9%), followed by *T. capilipes* (H6: 73.2%); while other ethanol extracts showed a low percentage chelating capacity in the range 0–42.9% compared with water extracts. Flavonoid and phenolic compounds are known to act as antioxidants, radical scavengers and metal chelators [27]. These extracts have appreciable amounts of flavonoid and phenolic compounds that may contribute to their chelating ability.

The antioxidant activities of the water and ethanol extracts measured by the FRAP assay are shown in Tables 3 and 4. A wide variation was observed. The water extract of *P. vulgaris* (H9) showed the highest FRAP (CPE 103.9 mg/g) while *P. grandiflora* (H13) showed the lowest FRAP (CPE 2.9 mg/g). The FRAP of the ethanol extracts was in the range 3.56–87.5 CPE mg/g (Table 4). FRAP was significant in some of the water and ethanol extracts owing to the presence of phenolic and flavonoid compounds and a similar trend is observed for many other plant extracts that have been studied [28]. The reducing properties are generally associated with compounds that can donate hydrogen atoms to free radicals and convert them into stable non-reactive molecules [29].

**Figure 2:**
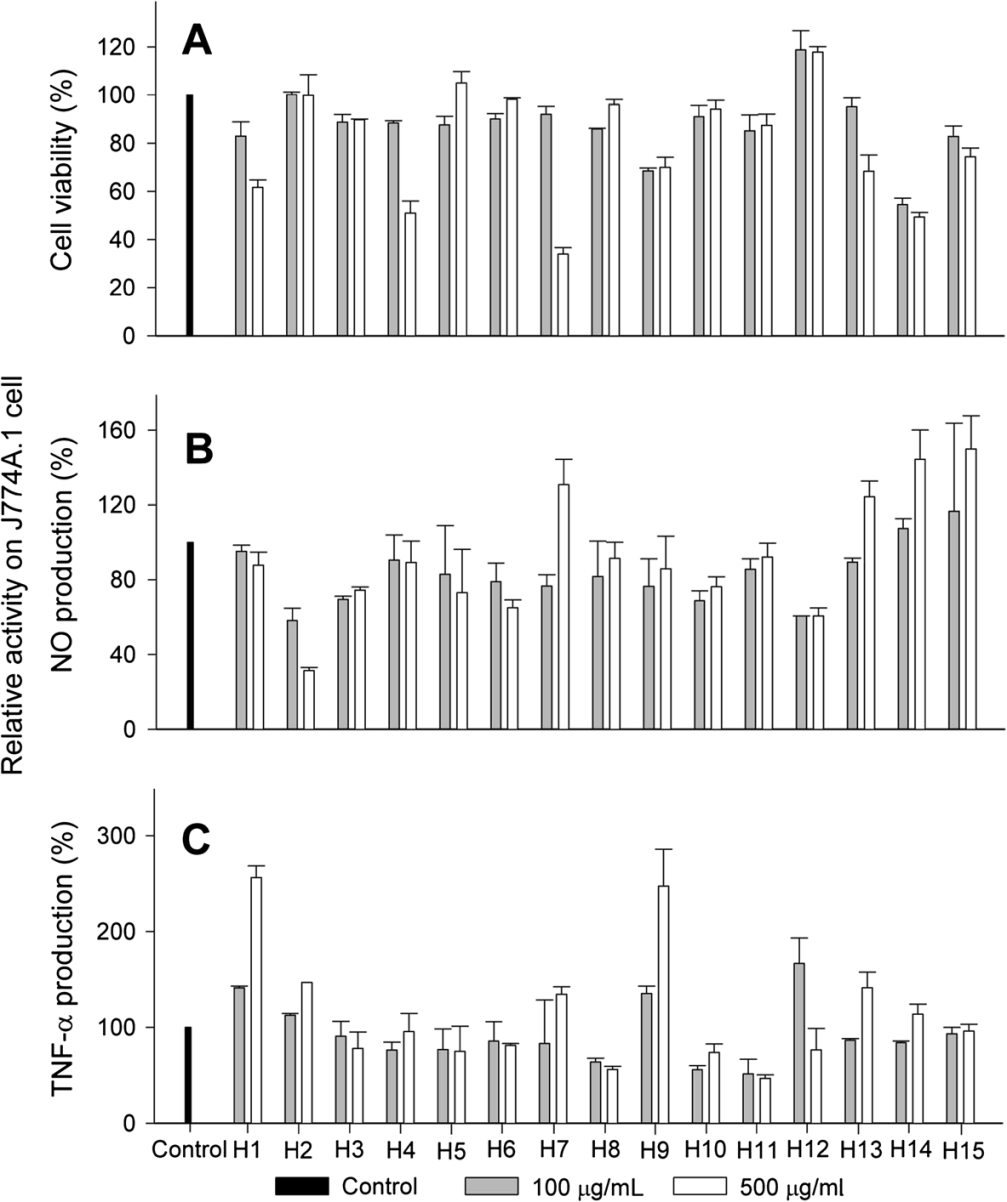
**Effect of water extracts of herbs on LPS-stimulated macrophage production of NO and TNF-α, and cell viability. A**: Cell viability. **B**: NO production. **C**: TNF-α production.

The anti-inflammatory properties of the water extracts were evaluated on the basis of their inhibition of NO and TNF-α production in LPS- and IFN-γ activated macrophages (Figure 2). NO is an important intracellular and intercellular regulator of multiple biological functions, including macrophage-mediated cytotoxicity, neurotransmission and smooth muscle relaxation [30,31]. Overexpression of NO has been associated with oxidative stress [32,33] and with the pathophysiology of various diseases such as arthritis, diabetes, autoimmune disease and chronic inflammation [34,35]. It has also been shown that the production of TNF-α is crucial for the synergistic induction of NO synthesis in IFN-γ- and LPS-stimulated macrophages [36,37]. The cell toxicity of the plant extracts was determined by MTT assay. The results indicated that most of the extracts showed significant cell viability at a concentration of 100 μg/mL (85% or higher), except for *P. vulgaris* (H9) and *E. brevicornum* (H14). Therefore the water extracts of these plants exhibited low toxicity. Most of the extracts (except for H7, H13, H14 and H15) also inhibited macrophage NO production (Figure 2B). H7, H13, H14 and H15 extracts activated macrophage NO production as well as TNF-α production (Figure 2C). The role of TNF-α production was suggested for the antitumor activity of phenols and flavonoids [38], but the underlying mechanisms by which different flavonoid and phenolic compounds affect TNF-α and NO production are to be investigated.

## Conclusion

The selected herbs could be a rich source of antioxidants and free radical scavenging compounds. The levels of phenolic and flavonoid compounds were correlated with the antioxidant and anti-inflammatory activities of the herb extracts.

## Abbreviations

NO: Nitric Oxide; TNF-α: Tumour Necrosis Factor-alpha; DPPH: 2,2-diphenyl-1-picrylhydrazyl; FRAP: Ferric reducing antioxidant power; LPS: Lipopolysaccharide; IFN-γ: Interferon gamma; DMEM: Dulbecco’s modified Eagle’s medium; FBS: Foetal bovine serum.

## Competing interests

The authors declare that they have no competing interests.

## Authors’ contributions

SRK, SCJ and CK designed the study and wrote the manuscript. PD, SCJ, SRK and SL performed the experiments. PD, SRK, SCJ, CK, and SL analysed the data. All authors read and approved the final manuscript.

## References

[B1] Cheeseman KH Slater TF An introduction to free radical biochemistry Br Med Bull 1993 49 481 493 8221017 10.1093/oxfordjournals.bmb.a072625

[B2] Ajith TA Janardhanan KK Indian medicinl mushrooms as a source of antioxidant and antitumor agents J Clin Biochem Nutr 2007 40 157 162 10.3164/jcbn.40.157 18398492 PMC2275760

[B3] Liu H Visner GA Zander DS, Popper HH, Jagirdar J, Haque AK, Cagle PT, Barrios R Oxidants and antioxidants Molecular Pathology of Lung Diseases 2008 Springer 470 475

[B4] Zhang L Ravipati AS Koyyalamudi SR Jeong SC Reddy N Smith PT Bartlett J Shanmugam K Münch DG Wu MJ Antioxidant and anti-inflammatory activities of selected medicinal plants containing phenolic and flavonoid compounds J Agr Food Chem 2011 59 12361 12367 10.1021/jf203146e 22023309

[B5] Tang SY Whiteman M Peng ZF Jenner A Yong EL Halliwell B Characterization of antioxidant and antiglycation properties and isolation of active ingredients from traditional Chinese medicines Free Radic Biol Med 2004 36 1575 1587 10.1016/j.freeradbiomed.2004.03.017 15182859

[B6] Cai Y Luo Q Sun M Corke H Antioxidant activity and phenolic compounds of 112 traditional Chinese medicinal plants associated with anticancer Life Sci 2004 74 2157 2184 10.1016/j.lfs.2003.09.047 14969719 PMC7126989

[B7] Dragland S Senoo H Wake K Holte K Blomhoff R Several culinary and medicinal herbs are important sources of dietary antioxidants J Nutr 2003 133 1286 1290 12730411 10.1093/jn/133.5.1286

[B8] Akinmoladun AC Obuotor EM Farombi EO Evaluation of antioxidant and free radical scavenging capacities of some Nigerian indigenous medicinal plants J Med Food 2010 13 444 451 10.1089/jmf.2008.0292 20192848

[B9] Özen T Çöllü Z Korkmaz H Antioxidant properties of Urtica pilulifera root, seed, flower, and leaf extract J Med Food 2010 13 1224 1231 10.1089/jmf.2009.1303 20828318

[B10] Hendra R Ahmad S Oskoueian E Sukari A Shukor MY Antioxidant, Anti-inflammatory and Cytotoxicity of Phaleria macrocarpa (Boerl.) Scheff Fruit BMC Complem Altern M 2011 11 110 10.1186/1472-6882-11-110 PMC3354343 22070850

[B11] Talhouk R Karam C Fostok S El-Jouni W Barbour E Anti-inflammatory bioactivities in plant extracts J Med Food 2007 10 1 10 10.1089/jmf.2005.055 17472460

[B12] Wang C Schuller Levis GB Lee EB Levis WR Lee DW Kim BS Park SY Park E Platycodin D and D3 isolated from the root of Platycodon grandiflorum modulate the production of nitric oxide and secretion of TNF-[alpha] in activated RAW 264.7 cells Int J Immunopharmacol 2004 4 1039 1049 10.1016/j.intimp.2004.04.005 15222978

[B13] Cicco N Lanorte MT Paraggio M Viggiano M Lattanzio VA Reproducible, rapid and inexpensive Folin-Ciocalteu micro-method in determining phenolics of plant methanol extracts Microchem J 2009 91 107 110 10.1016/j.microc.2008.08.011

[B14] Cai Y Sun M Xing J Corke H Antioxidant phenolic constituents in roots of Rheum officinale and Rubia cordifolia: structure-radical scavenging activity relationships J Agr Food Chem 2004 52 7884 7890 10.1021/jf0489116 15612771

[B15] Zhishen J Mengcheng T Jianming W The determination of flavonoid contents in mulberry and their scavenging effects on superoxide radicals Food Chem 1999 64 555 559 10.1016/S0308-8146(98)00102-2

[B16] Brand-Williams W Cuveleir ME Berset C Use of a free radical method to evaluate antioxidant activity Lebensm Wiss Technol 1995 28 25 30 10.1016/S0023-6438(95)80008-5

[B17] Wu MJ O’Doherty PJ Fernandez HR Lyons V Rogers PJ Dawes IW Higgins VJ An antioxidant screening assay based on oxidant induced growth arrest in Saccharomyces cerevisiae FEMS Yeast Res 2011 11 379 387 10.1111/j.1567-1364.2011.00726.x 21375688

[B18] Chua MT Tung YT Chang ST Antioxidant activities of ethanolic extracts from twigs of Cinnamomum osmophleum Bioresource Technol 2008 99 1918 1925 10.1016/j.biortech.2007.03.020 17478090

[B19] Oyaizu M Studies on product of browning reaction prepared from glucosamine Jpn J Nutr 1986 44 307 315 10.5264/eiyogakuzashi.44.307

[B20] Cui S Reichner JS Mateo RB Albina JE Activated murine macrophages induce apoptosis in tumor cells through nitric oxide-dependent or independent mechanisms Cancer Res 1994 54 2462 2467 8162595

[B21] Behl C Davis J Cole GM Schubert D Vitamin E protects nerve cells from amyloid beta protein toxicity Biochem Biophys Res Commun 1992 186 944 950 10.1016/0006-291X(92)90837-B 1497677

[B22] Zhang L Koyyalamudi SR Jeong SC Reddy N Smith PT Ananthan R Longvah T Antioxidant and immunomodulatory activities of polysaccharides from the roots of Sanguisorba officinalis Int J Biol Macromol 2012 51 1057 1062 10.1016/j.ijbiomac.2012.08.019 22944198

[B23] Duncan DB Multiple range tests for correlated and heteroscedastic means Biometrics 1957 13 164 176 10.2307/2527799

[B24] Petti S Scully C Polyphenols, oral health and disease: a review J Dent 2009 37 413 423 10.1016/j.jdent.2009.02.003 19303186

[B25] Gioti E Fiamegos Y Skalkos D Stalikas C Antioxidant activity and bioactive components of the aerial parts of Hypericum perforatum L. from Epirus, Greece Food Chem 2009 117 398 404 10.1016/j.foodchem.2009.04.016

[B26] Chang HF Yang LL Radical-scavenging and rat liver mitochondria lipid peroxidative inhibitory effects of natural flavonoids from traditional medicinal herbs J Med Plants Res 2012 6 997 1006

[B27] Mira L Fernandez MT Santos M Rocha R Florencio MH Jennings KR Interactions of flavonoids with iron and copper ions: a mechanism for their antioxidant activity Free Radic Res 2002 36 1199 1208 10.1080/1071576021000016463 12592672

[B28] Katalinic V Milos M Kulisic T Jukic M Screening of 70 medicinal plant extracts for antioxidant capacity and total phenols Food Chem 2006 94 550 557 10.1016/j.foodchem.2004.12.004

[B29] Gordon MH Hudson BJF The mechanism of antioxidant action in vitro Food antioxidants 1990 London: Elsevier Applied Science 1 18

[B30] Ignarro LJ Regulation of cytosolic guanylyl cyclase by porphyrins and metalloporphyrins Adv Pharmocol 1994 26 35 65 10.1016/s1054-3589(08)60050-2 7913618

[B31] Beckman JS Koppenol WH Nitric oxide, superoxide, and peroxynitrite: the good, the bad, and ugly Am J Physiol 1996 271 C1424 C1437 8944624 10.1152/ajpcell.1996.271.5.C1424

[B32] Sies H Mehlhorn R Mutagenicity of nitroxide-free radicals Arch Biochem Biophys 1986 251 393 396 10.1016/0003-9861(86)90087-1 3539021

[B33] Ji Y Akerboom TP Sies H Thomas JA S-nitrosylation and S-glutathiolation of protein sulfhydryls by S-nitroso glutathione Arch Biochem Biophys 1999 362 67 78 10.1006/abbi.1998.1013 9917330

[B34] Moncada S Palmer RM Higgs EA Nitric oxide: physiology, pathophysiology, and pharmacology Pharmacol Rev 1991 43 109 142 1852778

[B35] Arteel GE Briviba K Sies H Protection against peroxynitrite FEBS Lett 1999 445 226 230 10.1016/S0014-5793(99)00073-3 10094462

[B36] Green SJ Crawford RM Hockmeyer JT Meltzer MS Nacy CA Leishmania major amastigotes initiate the L-arginine-dependent killing mechanism in IFN-gamma-stimulated macrophages by induction of tumor necrosis factor-alpha J Immunol 1990 145 4290 4297 2124240

[B37] Jun CD Choi BM Kim HM Chung HT Involvement of protein kinase C during taxol-induced activation of murine peritoneal macrophages J Immunol 1995 154 6541 6547 7759887

[B38] Peng B Hu Q Sun L Chen Y Liu X Li J Chang Q Wang L Tang J Duchesnea phenolic fraction inhibits tumor growth through restoting the Th1/Th2 balance in U14 cervical cancer bearing mice Chin Med 2012 3 42 45 10.4236/cm.2012.31007

